# A discovery platform to improve visibility of linked data across Australia.

**DOI:** 10.23889/ijpds.v9i5.2596

**Published:** 2024-09-18

**Authors:** Emma Gearon, Bronwyn Wyatt, Ingrid Evans, Megan Fraser, Ann-Kristin Raymer, Fleur de Crespigny, Melanie Dunford, Claire Sparke

**Affiliations:** 1 Australian Institute of Health and Welfare

## Background

Metadata plays a crucial role in the health research infrastructure ecosystem. Despite the abundance of metadata for data collections in Australia, there is no simple way for researchers to search for standardised metadata about linked and linkable data collections across sectors or jurisdictions, making it an arduous and time-intensive task.

## Approach

The project comprised three phases: an initial scoping exercise to understand the current state of metadata and best practice; a national consultation involving researchers data linkage staff and data custodians to develop a high-fidelity prototype; and a final build and implementation phase. The platform underwent several prototyping and testing cycles to refine the digital experience.

## Results

Key expert interviews confirmed that there is a wealth of metadata available in Australia, but it is difficult for researchers to access and evaluate. Consultations with researchers identified opportunities to standardise metadata and provide a centralised platform to enhance the discoverability of linkable data collections for research. High value platform features included searching, browsing and filtering capabilities, variable list metadata, standardised formats, sample data, and frequently asked questions. The final design and functionality reflected user consultations and data custodian input on feasibility and resulted in the standardisation of metadata for linked data collections across sectors and jurisdictions.

## Conclusion

The Population Health Research Network developed and implemented a metadata platform to enable researchers to assess and evaluate the suitability of data collections for data linkage projects more readily. Improved metadata accessibility will save time and enhance the quality of linked data applications.

